# Supplementary material for: CRISPR-Cas13a-Based Assay for Accurate Detection of OXA-48 and GES Carbapenemases
Source: Microbiol Spectr. 2023 Jul 19;11(4):e01329-23. doi: 10.1128/spectrum.01329-23 (PMC10434040; doi:10.1128/spectrum.01329-23)
Supplement: Supplemental file 1 — Supplemental material. Download spectrum.01329-23-s0001.pdf, PDF file, 0.05 MB [file spectrum.01329-23-s0001.pdf]

SUPPLEMENTARY MATERIAL

Commercial kit	Technique	*bla*$_{GES}$ gene detection	Time	Sample / Isolate	Nucleic acid purification/ concentration	Sensitivity (%)	Specificity (%)	Price per reaction[a]	Ref
Eazyplex® SuperBug CRE	LAMP fluorescent	No	15 min	Isolate	No	100	100	€€€	(28)
LAMP-HNB	LAMP –HNB	No	1 h	Isolate	No	100	100	€	(29)
Check Direct CPE	Multiplex qPCR	No	<1.5 h	Sample	No	100	88	€€	(26)
Amplidiag CarbaR+MCR Kit	Multiplex qPCR	Yes	3 h	Sample	Yes	92.5	100	€€	(21)
EntericBio CPE	Multiplex qPCR	Yes	3 h	Isolate	No	100	100	€€	(22)
BD Max System	Multiplex qPCR	Yes	<1 h	Isolate	Yes	100	100	€€€	(23)
In-house qPCR	Multiplex qPCR	No	<1.5 h	Isolate	No	100	100	€€*	(27)

In-house qPCR	Multiplex qPCR	Yes	1 h	Isolate	Yes	100	100	€€	(24)
In-house qPCR	Multiplex qPCR	Yes	3 h	Isolate	Yes	100	100	€€*	(25)
Luminex xTAG®	Multiplex PCR + Luminex	Yes	5 h	Isolate	Yes	100	99.4	€€	(20)
In-house PCR	Multiplex PCR + electrophoresis	Yes	<2 h	Isolate	No	100	100	€	(19)
Multiplex microarray	Multiplex microarray	No	2 h	Sample	No	98.3	99.6	€€€	(30)

Supplementary Table S1. Percentages of sensitivity and specificity parameters reported in 12 articles on the application of genotypic methods for the detection of OXA-48 and GES carbapenemase genes.

[a]Prices per reaction (under 10 € = €, 10 € to 49 € = €€, and 50 € to100 € = €€€) from the UK Health Security Agency, regardless of the price of the equipments.

(*) Standard real time PCR price (33).
